# The Role of Cytology in the Diagnosis of Subcentimeter Thyroid Lesions

**DOI:** 10.3390/diagnostics11061043

**Published:** 2021-06-06

**Authors:** Vincenzo Fiorentino, Marco Dell’ Aquila, Teresa Musarra, Maurizio Martini, Sara Capodimonti, Guido Fadda, Mariangela Curatolo, Emanuela Traini, Marco Raffaelli, Celestino Pio Lombardi, Alfredo Pontecorvi, Luigi Maria Larocca, Liron Pantanowitz, Esther Diana Rossi

**Affiliations:** 1 Division of Anatomic Pathology and Histology, Fondazione Policlinico Universitario “Agostino Gemelli”-IRCCS, 00168 Rome, Italy; vins8966@gmail.com (V.F.); mzrk07@gmail.com (M.D.A.); teresa.musarra@guest.policlinicogemelli.it (T.M.); maurizio.martini@policlinicogemelli.it (M.M.); saracapodimonti@libero.it (S.C.); guido.fadda@unime.it (G.F.); mariangela.curatolo@guest.policlinicogemelli.it (M.C.); luigimaria.larocca@unicatt.it (L.M.L.); 2 Division of Endocrine Surgery, Fondazione Policlinico Universitario “Agostino Gemelli”-IRCCS, 00168 Rome, Italy; emanuela.traini@policlinicogemelli.it (E.T.); marco.raffaelli@policlinicogemelli.it (M.R.); celestinopio.lombardi@policlinicogemelli.it (C.P.L.); 3 Division of Endocrinology-Fondazione, Policlinico Universitario “Agostino Gemelli”-IRCCS, 00168 Rome, Italy; alfredo.pontecorvi@policlinicogemelli.it; 4 Department of Pathology & Clinical Labs, University of Michigan, Ann Arbor, MI 48103, USA; lironp@med.umich.edu

**Keywords:** fine needle aspiration, thyroid nodules, Bethesda thyroid classification system, subcentimeter nodules, personalized medicine, cancer

## Abstract

Thyroid nodules are common and typically detected by palpation and/or ultrasound (US). Guidelines have defined the management of large nodules, but controversy exists regarding nodules ≤ 1 cm. We evaluated a cohort of patients with subcentimeter nodules to determine their rate of malignancy (ROM). A total of 475 thyroid FNAs of lesions ≤ 1 cm with available follow-up were identified from January 2015–December 2019. For comparative analysis, we added a control series of 606 thyroid lesions larger than 1 cm from the same reference period. All aspirates were processed with liquid-based cytology and classified according to The Bethesda System for Reporting Thyroid Cytopathology (TBSRTC). Subcentimeter nodules were stratified as 35 category I—non-diagnostic cases (ND; 7.3%), 144 category II—benign lesions (BL; 30.3%), 12 category III—atypia of undetermined significance/follicular lesion of undetermined significance (AUS/FLUS; 2.5%), 12 category IV—follicular neoplasm/suspicious for follicular neoplasm (FN/SFN; 2.5%), 124 category V—suspicious for malignancy (SM; 26.1%), and 148 category VI—positive for malignancy (PM; 31.1%). A total of 307 cases (64.6%) underwent subsequent surgery. Only one ND and three BLs had a malignant outcome. ROM for indeterminate lesions (III + IV) was 3.2%; with 1.6% for category III and 3.2% for category IV. ROM for the malignant categories (V + VI) was 88.2%. The control cohort of lesions demonstrated a higher number of benign histological diagnoses (67.3%). We documented that 57.2% of suspected subcentimeter lesions were malignant, with a minor proportion that belonged in indeterminate categories. There were very few ND samples, suggesting that aspirates of subcentimeter lesions yield satisfactory results. Suspected US features in subcentimeter lesions should be evaluated and followed by an interdisciplinary team for appropriate patient management.

## 1. Introduction

Thyroid nodules are commonly found in both pediatric and adult patients. Despite the fact that the majority of thyroid nodules are benign, the incidence of thyroid carcinoma, especially in the USA, has increased more than any other cancer [1,2,3,4,5]. This increase is due to a rise in the preoperative evaluation of the thyroid gland, incidental ultrasound diagnoses of subcentimeter carcinomas, and greater diagnoses of follicular variants of papillary thyroid carcinoma (FVPTC) [6,7,8,9,10,11,12,13].

The number of papillary microcarcinomas (equal to or smaller than 1 cm) that have been diagnosed has doubled, whilst the number of subcentimeter (below 1 cm) thyroid nodules diagnosed as FVPTC has tripled [1,6,10]. The increased number of subcentimeter carcinomas being reported in histological samples seems to be due to the high number of small lesions being detected preoperatively, which are frequently found as incidental lesions, especially with ultrasound (US). The correct cytological diagnosis of subcentimeter lesions is critical to determine their subsequent clinical management. Regardless of their size, fine needle aspiration cytology (FNAC) is a useful means to correctly diagnose more than 70% of thyroid nodules [14,15,16,17,18,19,20,21,22]. Thus, it is important to have stringent diagnostic criteria for FNAC samples, even for subcentimeter thyroid lesions, in order to correctly triage those that turn out to have cancer and avoid the overtreatment of indolent lesions. Guidelines in the literature have offered recommendations for the biopsy of nodules larger than 1 cm, leaving nodules smaller than 1 cm to the discretion of clinicians and/or radiologists [23,24,25]. Specifically, the American Thyroid Association (ATA) guideline in 2006 recommended that only nodules of potential clinical risk and larger than 1 cm should be assessed using FNAC [24].

In 2009, the revised ATA guidelines suggested that even 5 to 10 mm nodules with suspicious US features (hypoechogenicity, microcalcifications, infiltrative margins, increased vascularity, and nodules that have a taller-than-wide shape) should undergo FNAC [25]. However, for subcentimeter nodules with suspicious US features, their malignancy rate is uncertain [26,27,28,29,30,31,32,33,34,35]. Ding et al. reported that, using US features, they were able to stratify subcentimeter nodules into two groups, each with a different ROM [27].

Several studies have demonstrated that a certain subset of microcarcinoma can behave aggressively, showing capsular invasion, multifocality, and lymph node metastases [1,2,3,4,5,6,7,8,9,10,11,19]. As a result, several microcarcinomas are consequently treated as classical carcinomas. The cytological evaluation of subcentimeter nodules, in order to either recognize malignancy or identify a benign subcentimeter lesion, is critical and likely will contribute to the correct discrimination between benign and malignant subcentimeter thyroid nodules. The diagnosis of indeterminate categories for subcentimeter lesions may lead to some uncertainty for correct follow-up and a rationale for active surveillance for these small nodules.

The aim of this study was to examine a cohort of patients with suspected US subcentimeter thyroid lesions who underwent FNAC and report on their cytopathologic and histological follow-up findings, supported also by ancillary techniques (immunocytochemistry and molecular testing). Furthermore, a series of equal to and larger than 1 cm thyroid lesions was compared with the study group.

## 2. Materials and Methods

Institutional (Catholic University of the Sacred Heart) ethical approval was received for this study on January 2021, approval internal protocol number 60321. A retrospective search was performed for all thyroid FNAC cases diagnosed during a five-year period (January 2015 to December 2019) at the Catholic University Agostino Gemelli Hospital in Rome, Italy. The electronic medical record system Armonia-Metafora, Italy (CU), was also searched for thyroidectomy specimens during the same study period. Patient age, gender, FNAC diagnoses, US features, and follow-up information were recorded. All available pathology slides were reviewed. The majority of these thyroid nodules were evaluated and biopsied under US guidance by clinicians and radiologists.

### 2.1. Thyroid FNAC Specimens

All aspirations (usually with two passes performed for each lesion) were performed with 25 to 27 G needles. No rapid on-site assessment for adequacy of material was performed. All patients consented to their procedure with written informed consent and the study protocol was approved by the institute’s committee on human research. All FNAC specimens were processed using a ThinPrep 5000^TM^ processor (Hologic Co., Marlborough, MA, USA). Prepared slides were fixed in 95% methanol and stained with a Papanicolaou stain. Residual aspirated material was stored in Preservcyt solution for potential ancillary studies, including immunocytochemistry (ICC) and molecular testing [20].

Specimen adequacy was determined following The Bethesda System for Reporting Thyroid Cytology (TBSRTC) [35]. Cytology cases were classified and diagnosed according to the new Italian Working Group SIAPEC-IAP classification [36]. All cases were re-evaluated and then re-classified according to the second edition of TBSRTC [35]. For the purposes of this study, analyses were conducted and reported using TBSRTC terminology. During the study period the following distribution of cytologic diagnoses were recorded: 5.9% non-diagnostic (ND) cases (including those with cyst contents only), 77.8% benign lesions (BL), 3% with atypia of undetermined significance/follicular lesion of undetermined significance (AUS/FLUS), 6.1% follicular neoplasms (FN), 2.2% SFM, and 5% malignant (M) cases. All cytology and histology cases were reviewed by two cytopathologists (EDR and GF) whilst the re-classification according to TBSRTC was done by one cytopathologist (EDR). Cases with an equivocal interpretation were subject to consensus review. The concordance between SIAPEC-IAP and TBSRTC classification systems was 95.9%.

For the purpose of this study, we selected all 475 subcentimeter lesions identified during the reference period, including 307 with histological follow-up. As per institutional agreement, all FNACs were performed by endocrinologists and endocrine surgeons under sonographic guidance for the following reasons: (i) iso-hypoechoic pattern, (ii) lesions with infiltrative margins, (iii) family history of PTC, (iv) personal history of head and neck irradiation, and (v) nodules with suspicious lymph nodes based on neck US. Furthermore, we compared the subcentimeter series with a cyto-histological control series of equal to or larger than 1 cm thyroid lesions from the same reference period.

### 2.2. Ultrasound Evaluation

**Figure 1:**
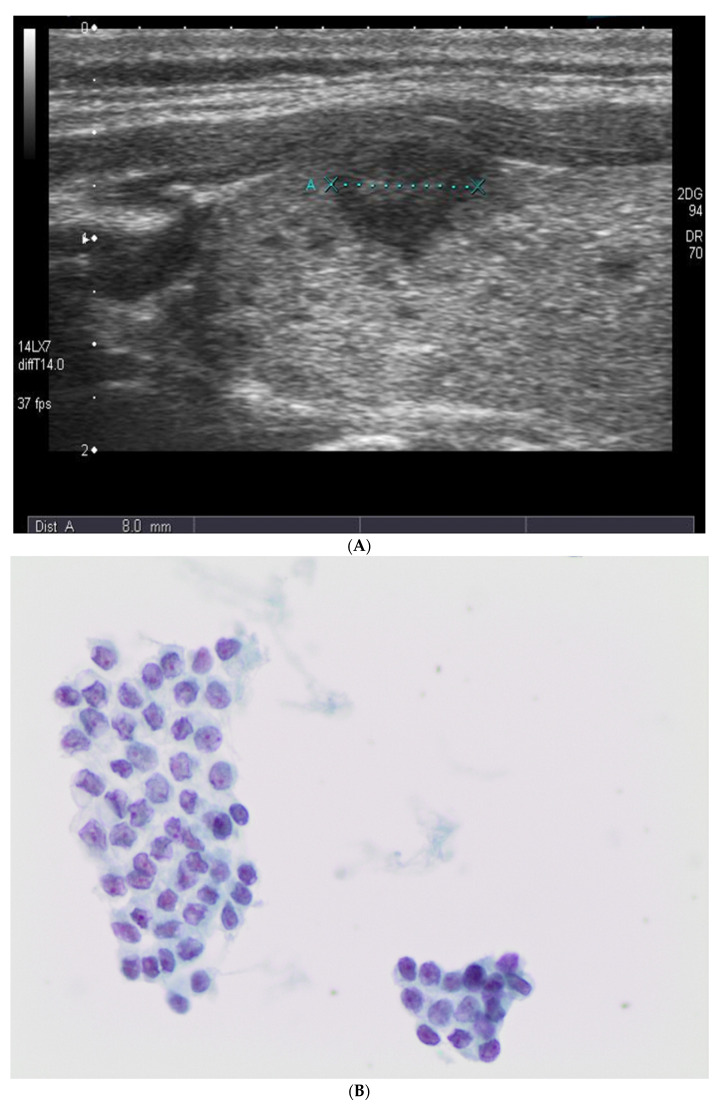

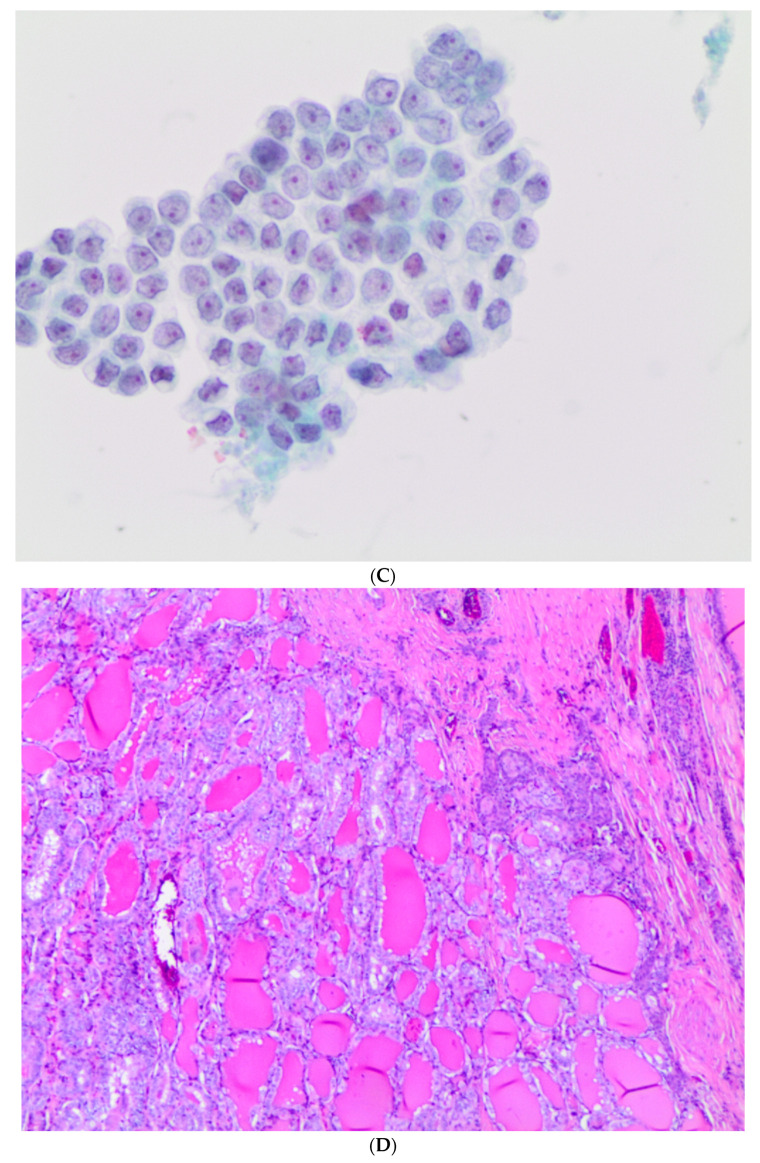
(**A**) Ultrasound picture from a subcentimeter lesion that underwent fine needle aspiration cytology. (**B**,**C**) The picture shows the morphological features diagnostic for a suspicious malignancy characterized by atypical nuclei with some grooves in absence of univocal features of carcinoma (200× and 400× liquid-based cytology (LBC), respectively). (**D**) The picture shows the histological features from the same case diagnosed as a follicular variant of papillary thyroid carcinoma (FVPTC) (200×, H&E).

US images were obtained using 5–12 MHz linear transducers (HDI 5000 and IU-22, Philips). Real-time US was performed by 5 different endocrinologists and endocrine surgeons with radiological experience ranging between 5–25 years, often accompanied by fellows and residents. US features of all thyroid nodules that underwent US-guided FNAC were prospectively recorded according to their internal composition, echogenicity, margin calcification, shape, and vascularity of thyroid lesions adopting the criteria suggested by the TI-RADS system score [37]. For internal composition, we differentiated solid, cystic, and mixed lesions. The echogenicity of nodules was classified as hyperechogenic, hypoechogenic (shown in Figure 1A), and isoechogenic. The margins were divided into lesions that were well-circumscribed, microlobulated, and irregular–infiltrative. Calcifications were subclassified as micro- and/or macro-calcifications. For the shape of the nodules, we documented that they exhibited a “taller-than-wide” shape. Vascularity was classified as peripheral, central, both peripheral and central, and no vascularity.

### 2.3. Immunocytochemistry (ICC) Analysis

We performed ICC on all liquid-based cytology (LBC) samples. HBME-1 and galectin-3 ICC was performed using a protocol previously described by our group [38,39]. The minimal percentage of adequate lesional cells for the performance of ICC evaluation was defined at 30% in LBC samples. Lesional cells were interpreted to be positive when at least 50% of the cells demonstrated strong cytoplasmic staining. To avoid false negative and/or false positive yields, this 50% ICC cutoff value was also used for histological tissue sections. A case was considered to be overall positive for malignancy when there was concomitant expression of both immunomarkers. Adequate galectin-3 immunoreactivity was represented by cytoplasmic staining, and suitable HBME-1 staining was determined by immunoreactivity within the cytoplasm with accentuation on the cytoplasmic membrane and within the lumen. Positive controls included mesothelioma for HBME-1 (membranous positivity) and histiocytes for galectin-3 (cytoplasmic staining). Lymphocytes identified in the majority of the thyroid slides were used as a negative control.

### 2.4. Histology Specimens

All surgical specimens were fixed in 10% buffered formaldehyde, embedded in paraffin, and 5 micron-thick sections then stained with hematoxylin and eosin (H&E). The diagnosis of classical variant of papillary thyroid carcinoma (PTC) was based on the presence of true papillary structures and distinctive nuclear features (e.g., nuclear clearing, grooves, pseudoinclusions). The diagnosis of follicular variant of PTC (FVPTC) relied upon the detection of follicular architecture coupled with the nuclear features of PTC in multiple foci. Encapsulated tumors with either lympho-vascular invasion (within the capsule or beyond) or capsular penetration were diagnosed as invasive FVPTC. The different PTC variants were classified according to the WHO 2017 criteria [40]. For the definition of tall cell variant (TCV) of PTC, we included cases of PTC with an equal or more than 30% TCV component [41]. The histological diagnosis of noninvasive follicular thyroid neoplasm with papillary like nuclear features (NIFTP) was rendered according to the criteria described by Nikiforov et al. [42]. All malignant cases were classified according to the eighth edition of the tumor–node–metastasis (TNM)-based staging system recommended by the American Joint Commission on Cancer (AJCC) [41]. The lymph nodes were resected based on the macroscopic finding during surgery.

### 2.5. Molecular Analysis for BRAF and TERT Mutation

DNA was extracted from both LBC stored material and paraffin-embedded tissues. For cytological samples, all AUS/FLUS and FN/SFN cases, regardless of size, were analyzed with the use of ICC and molecular testing.

*BRAFV600E* mutational analysis was performed on DNA extracted from cytological and surgical specimens containing at least 70% tumor. Details of the molecular protocol employed have been previously published by our group [43]. For *hTERT*, genomic DNA was extracted from LBC samples stored in PreservCyt solution (Hologic, Marlborough, MA, USA) with the QIAamp DNA mini kit (Qiagen, Hilden, Germany), according to the manufacturer’s protocol. PCR was performed in 20 μL reactions containing genomic DNA (100 ng), 0.2 μmol/L of primers (forward: 5′-CACCCGTCCTGCCCCTTCACCTT-3′ and reverse: 5′-GGCTTCCCACGTGCGCAGCAGGA-3) and 2× PCRBIO HS Taq Mix (PCR Biosystems Inc., Wayne, Pennsylvania USA). PCR conditions were as follows: initial denaturation at 95 °C for 10 min, followed by 35 cycles at 95 °C for 40 s, 62 °C for 40 s, and 72 °C for 40 s. *hTERT* promoter amplification was performed on an C1000 Touch Thermal Cycler (BioRad, Hercules, CA, USA). The fragment was separated by electrophoresis on 2% agarose gels containing ethidium bromide and visualized by UV illumination. The PCR product was treated with EXOSap (UBS, Sial, Rome, Italy), following the manufacturer’s protocol, and directly sequenced using a BigDye Terminator kit v3.1 (Applied Biosystem, Foster City, CA, USA) with forward and reverse primers in an ABI PRISM 3100 Genetic Analyzer (Applied Biosystems).

### 2.6. Statistical Analysis

Statistical analysis was performed using GraphPad Prism 5 software (GraphPad Software, San Diego, CA, USA) and MedCalc v. 10.2.0.0 (MedCalc Software, Mariakerke, Belgium). The statistical comparison of continuous variables was performed using the Mann–Whitney *U*-test or paired *t*-test, as appropriate. The comparison of categorical variables was performed using the chi-square statistic and Fisher’s exact test. *p*-values less than 0.05 were considered as statistically significant.

## 3. Results

The entire series of subcentimeter lesions included 475 cytology samples examined during the study period. All subcentimeter lesions were discovered incidentally during radiologic screening for causes either unrelated to the thyroid gland or in the context of a well-known history of a nodular thyroid gland. The patient demographics and clinical/pathologic features of these individuals are provided in Table 1. There were 347 (73%) cytological samples (that were smaller than 1 cm) for which there was histological follow-up. The series included 146 male and 329 female patients with a median age of 44.38 years (range: 16–81 years; mean: 46 years). Thyroid lesions ranged in size from 0.3 to 1 cm (Table 1). The data clearly documented that there was no significant difference in the size of lesions among the different diagnostic categories. No notable statistical correlation was found for patients with subcentimeter thyroid lesions with respect to recorded clinical data. In our series, we had only 15 cases out of 307 (4.9%) lesions with lymph node metastases (Table 1). Combining our data with the last edition of the AJCC TNM staging system, we had 276 malignant cases classified as stage I and 15 cases classified as stage II, confirming the indolent nature of the majority of microcarcinomas.

Table 1 also shows the data from control cases, which included larger than 1 cm lesions. Specifically, we had 606 cases with 386 surgical follow-up. The data, reported in Table 1, clearly documented that there was no significant difference in the size of lesions among the different diagnostic categories. No notable statistical correlation was found for patients with larger thyroid lesions with respect to recorded clinical data. We found only 46 cases out of 386 (12%) lesions with lymph node metastases; correlation with the AJCC TNM staging system demonstrated that 291 were stage I and 95 were stage II.

Comparing the two series, we had a higher number of benign diagnoses in the equal to or larger than 1 cm series, showing a significant cyto-histological correlation in benign diagnoses (*p* < 0.0001). Additionally, more lesions were diagnosed as indeterminate proliferations (AUS/FLUS and/or FN/SFN) in the second control group than in the group of subcentimeter nodules. The following distribution of thyroid diagnoses were reported for FNAC cases in the subcentimeter series: 35 ND, 144 BL, 12 AUS/FLUS, 12 FN/SFN, 124 SFM, and 148 M (Table 1). In Table 1 the yields for the equal to or larger than 1 cm nodules resulted in 29 ND, 226 BL, 54 AUS/FLUS, 142 FN/SFN, 48 SFM, and 107 M cases (Table 1).

Table 2 illustrates details of the thyroid nodules classified by TBSRTC and US pattern. Whilst the majority of nodules had a hypoechoic pattern, echogenicity did not demonstrate statistical significance in our subcentimeter series (*p* = 0.0562), nor in larger nodules. However, an irregular and infiltrative pattern combined with hypoechogenicity was an US feature that triggered a FNAC. All (100%) thyroid nodules had a peripheral perivascular component. The taller-than-wide feature was only found in 20 cases, which were all subsequently diagnosed as malignant (Table 2). For subcentimeter lesions, Fisher’s exact test allowed us to confirm the positive association between this echographic feature and malignancy (*p* value < 0.001) whilst the same comparison for the larger nodules was not statistically significant (*p* = 0.0092). Table 2 confirmed that the majority of nodules were hypoechoic (430 cases), followed by an isoechoic pattern in 164 cases, with only two cases showing a taller-than-wider US pattern (Table 2).

**Figure 2:**
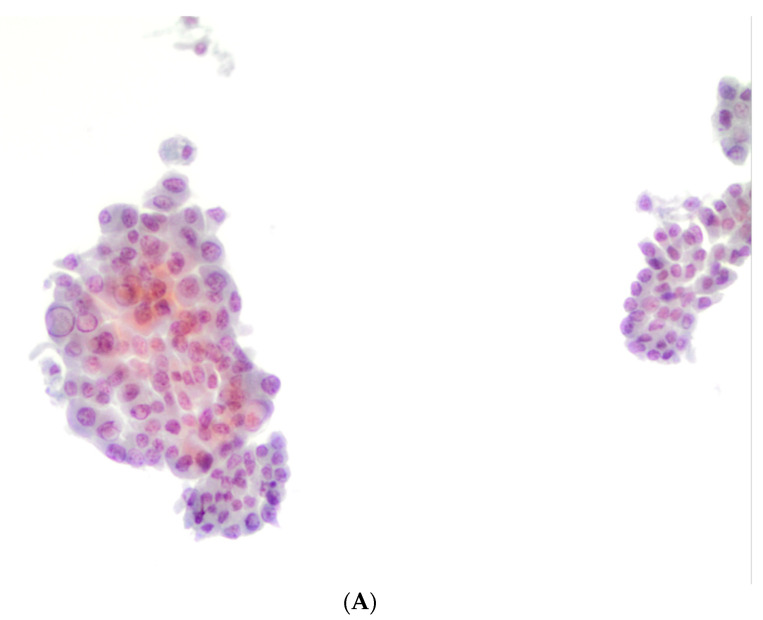

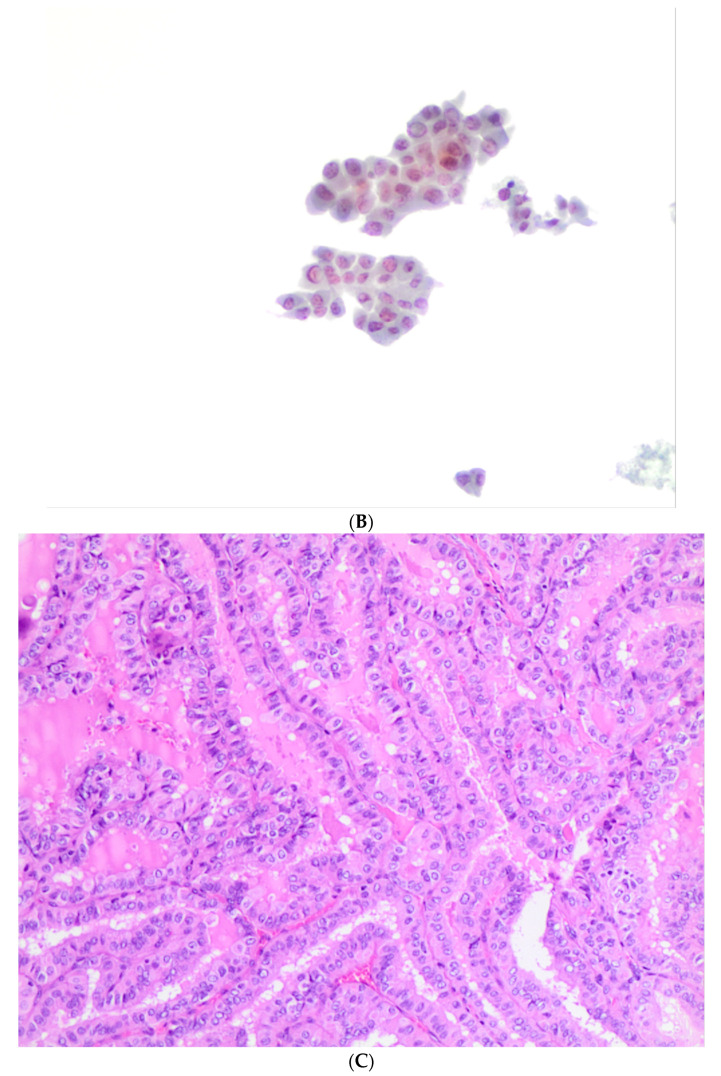
(**A**,**B**) The pictures show the morphological features diagnosed as positive for malignancy, favoring a papillary thyroid carcinoma (PTC) characterized by atypical and pleomorphic nuclei, some grooves, and nuclear pseudoinclusions (400× liquid-based cytology (LBC)). (**C**) The picture shows the histological features from the same case diagnosed as PTC with tall cell features (200×, H&E).

Table 3 summarizes the histological diagnoses that were rendered in 307 subcentimeter cases, which were comprised of 16 (5.2%) benign and 291 (94.7%) malignant lesions. Surgical follow-up stratified according to different cytological categories is shown in Table 3. The two ND cases (out of 35) that underwent surgery were diagnosed as either goiter or classical PTC. For 10 (out of 144) benign cases that had surgical follow-up, they were diagnosed as goiter (*n* = 5), follicular adenoma (FA) (*n* = 2), and PTC (*n* = 3). The 10 AUS/FLUS cases were diagnosed as Hashimoto thyroiditis (*n* = 2), FA (*n* = 3), PTC (*n* = 1) and invasive (I) FVPTC (*n* = 4). The 12 FN/SFN cases that had surgery were due to FA (*n* = 2), PTC (*n* = 4), hobnail variant of PTC (*n* = 2), and invasive (I) FVPTC (*n* = 4). The 124 SFM (shown in Figure 1B,C) cases included 1 FA, 103 PTC cases (shown in Figure 1D), 6 TCV-PTC, 2 hobnail variant of PTC and 10 invasive (I) FVPTC cases. The 148 M (shown in Figure 2A,B) cases were diagnosed as 119 PTC cases, 5 TCV-PTC (shown in Figure 2C), 3 Warthin-like PTC, 5 solid variant of PTC, 2 hobnail PTC, 10 invasive (I) FVPTC cases and 4 as medullary thyroid carcinoma (MTC). None of our cases had a histological diagnosis of NIFTP.

Table 4 summarizes the histological diagnoses that were rendered in 386 equal to or larger than 1 cm cases, which were comprised of 261 (67.6%) benign and 125 (32.3%) malignant lesions. Surgical follow-up stratified according to different cytological categories is shown in Table 4. The four ND cases (out of 29) that underwent surgery were diagnosed as either three goiters or one FVPTC. For 182 (out of 226) benign cases that had surgical follow-up, they were diagnosed as goiter (*n* = 145), FA (*n* = 31), Hashimoto thyroiditis (HT) (*n* = 3), PTC (*n* = 1), FVPTC (*n* = 1) or follicular carcinoma (FC) (*n* = 1). The 37 out of 54 AUS/FLUS cases were diagnosed as goiter (*n* = 4), HT (*n* = 1), or FA (*n* = 32). The 46 out of 142 FN/SFN cases that had surgery were due to goiter (*n* = 5), HT (*n* = 1), FA (*n* = 33), NIFTP (*n* = 2), FVPTC (*n* = 1), FC (*n* = 3), or oxyphilic follicular carcinoma (OFC) (*n* = 1). The 15 out of 48 SFM cases included 1 FA, 12 PTC cases, 1 TCV PTC and 1 I-FVPTC cases. The 102 out of 107 M cases were diagnosed as 89 PTC cases, 3 TCV PTC, 1 Warthin-like PTC, 2 hobnail PTC, 1 columnar cell variant of PTC (CCV PTC), and 6 as medullary thyroid carcinoma (MTC). Two of our cases had a histological diagnosis of NIFTP (Table 4).

For subcentimeter nodules, the concordance based upon cytological/histological correlation is demonstrated in Table 5. These findings show that an FNAC diagnosis of SFM and M was confirmed by histopathologic examination in 99.6% (271/272) of cases. Concerning the indeterminate categories of AUS/FLUS and FN/SFN, we confirmed that they each represented 2.5% of our series. In these indeterminate cases, we found a higher risk of malignancy (ROM) compared with that reported in TBSRTC, probably due to the high number of surgical follow-ups for those categories. One ND case resulted in a PTC diagnosis based upon histology, whilst three such cases in the benign category (out of 10 with surgical follow-up) were diagnosed as PTC (27.2%). The majority of AUS/FLUS cases underwent surgery (10/12, 83.3%) and showed an equal (50%) distribution between subsequent benign and malignant histopathology. Ten out of 12 FN cases had a histological diagnosis of carcinoma based upon histopathology (83.3%). The SFM category of cases was confirmed to have high ROM, as only 1 out of 128 of these cases had a subsequent histopathology diagnosis of FA (99%). All 148 cases with a preoperative cytological M diagnosis were confirmed to be malignant by histopathology, indicating that malignant lesions can be readily diagnosed by FNAC, irrespective of nodular size.

Table 6 summarizes the discordant diagnoses for the equal to or larger than 1 cm series. Only 12 cases had a discordant diagnosis in this subset of cases with larger nodular thyroid lesions. Specifically, one ND case resulted in a malignant diagnosis of FVPTC; 3 out of 182 benign cases resulted in a malignant diagnosis, including FC and PTC and its variants. Seven FN/SFN cases subsequently resulted in two NIFTP and five malignant lesions. One SFM case resulted in a histopathological diagnosis of FA. All the surgically removed malignant nodules (*n* = 102) had a histopathological malignant diagnosis.

For statistical purposes, we considered AUS/FLUS and FN/SFN to represent the “benign” group versus a second “malignant” group of patients comprised of SFM plus M cases. We also evaluated the diagnostic concordance between cytological and histological diagnoses, where AUS/FLUS was considered to represent “benign” whilst FN/SFN, together with SFM and M categories, were clustered together in a second “neoplastic” group, showing a significant *p* value < 0.001 calculated with Fisher’s exact test.

For the subcentimeter study group of cases, we calculated a sensitivity of 94.76%, specificity of 99.35%, positive predictive value (PPV) of 99.65%, and negative predictive value (NPV) of 90.65%. The same evaluation performed for the second control group of larger thyroid lesions resulted in 98.7% sensitivity, 95.8% specificity, 94% PPV, and 99% NPV.

In order to refine the indeterminate diagnoses (Table 7 and Table 8), all AUS/FLUS and FN/SFN cases, regardless of size, were analyzed with the use of ICC and molecular testing. ICC was carried out in 122 out of 475 cases for the subcentimeter nodules (25.6%, Table 7). For AUS/FLUS cases, ICC was supportive in four out of five cases with malignant histological diagnoses; the fifth case expressed positivity for HBME-1 only. The remaining AUS/FLUS cases that exhibited benign histopathology included two cases with positivity of HBME-1 only and three cases with a negative ICC panel. In the FN/SFN category, those cases with malignant histopathology (10 cases) had positivity for at least one immunomarker (HBME-1), and in four of them, both stains were positive. Two cases of FA were negative for the ICC panel on both cytology and histology specimens. Our four MTC cases were confirmed by calcitonin staining and histopathological diagnosis. For the SFM category, only 45 cases had ICC showing a concordant positive panel in 42 out of 45 cases (93.3%). The three remaining cases were found to have discordance in the expression of these antibodies, with expression of HBME-1. For the M category, we performed ICC in 14 cases, including four MTC cases positive for calcitonin and the remaining 10 M cases with a positive concordant ICC panel.

Table 8 shows the application of ICC for the equal to or larger than 1 cm group of thyroid nodules, including 122 out of 386 cases (31.6%). Three out of 37 AUS/FLUS cases had a concordant positive panel, which was associated with a histopathological malignant diagnosis. Eight AUS/FLUS cases showed a discordant immunopanel with positivity for HBME-1 only. For the FN/SFN cases, we found that the one FVPTC case had a concordant positive immunopanel. In the SFM cases, the one case histologically diagnosed as FA had a negative concordant immunopanel.

Molecular test results for *BRAF^V600E^* and *TERT* promoter mutations are shown in Table 9 and Table 10. Molecular testing showed 100% wild type (wt) *BRAF^V600E^* and *TERT* promoter mutations in AUS/FLUS cases, and 1/12 (8.3%) of FN/SFN cases with a mutated *BRAF^V600E^*. A minority of SFM and M samples had molecular testing, showing a small percentage of mutated cases in the three classical PTC and three TCV PTC cases. As reported in Table 9, all AUS/FLUS cases were *BRAF* wild type. A malignant diagnosis of FVPTC in the FN/SFN cases was confirmed by a *BRAF* mutation. The majority of SFM and M cases underwent molecular testing with only eight mutated cases out of 45 tested samples.

## 4. Discussion

The evaluation of thyroid nodules has increased since the widespread introduction of US-guided FNAC [10,11,12,13,14]. This has resulted in an increase in FNAC of incidentally detected, impalpable (including subcentimeter) thyroid lesions. US-guided FNAC offers a reliable, simple, and cost-effective method with minimal side effects to assess thyroid nodules. Whilst FNAC is extremely useful in the management of thyroid lesions larger than 1 cm, its role in evaluating subcentimeter lesions is still debated, albeit the revised ATA guidelines, which suggest that even 5 to 10 mm nodules with suspicious US features (including hypoechogenicity, microcalcifications, infiltrative margins, increased vascularity, and nodules that have a taller-than-wide shape) are likely to undergo FNAC [25]. Our described series underwent FNAC, following the ATA guidelines [23], only in subcentimeter cases with suspicious features on US, and/or for those incidentally detected lesions, often linked with patient psychological stress, and/or a family history of thyroid carcinomas and/or head and neck irradiation. As expected, in the current study, subcentimeter nodules with infiltrative margins and a taller-than-wide shape were associated with a high ROM (88.5%). Of note, all SFM and M cases showed irregular and infiltrative margins, with 20 of them exhibiting a taller-than-wide shape. These data are aligned with that reported by Chng et al. and Maia et al., who convey that irregular and infiltrative margins on US are strong predictors for malignancy [11,26]. Ding et al. reported that, using US features, they were able to stratify subcentimeter nodules into two groups, each with a different ROM [27]. They reported that patients with two or more predictors on US had a 74.2% ROM, whilst patients with none or only one predictor had a 20.4% ROM. One of the most controversial US features seems to be hypoechogenicity [44,45,46]. In our series, the majority of lesions had a hypoechoic pattern. Whilst this US finding was relevant in the decision to perform US-guided FNAC, hypoechogenicity was not itself a statistically significant parameter related to diagnosis in our series. The same result was evident for the second group of equal to or larger than 1 cm lesions characterized by the fact that benign and malignant nodules were mostly hypoechoic and isoechoic on US evaluation. The results confirmed that some US features, regardless of nodular size, need to be carefully identified as is often seen in suspicious and malignant lesions.

One may anticipate greater difficulty in obtaining adequate diagnostic material with very small thyroid lesions. Indeed, Mazzaferri and Sipos reported a high rate of ND cytological results in subcentimeter nodules [10]. On the other hand, Koo et al. reported a sensitivity and specificity of 96.8% and 100%, respectively, in their patients’ subcentimeter thyroid nodules [10]. We did not find a significant decrease in FNAC adequacy or accuracy related to sampling smaller sized nodules. Instead, in our study, a diagnostic rate of 93% was achieved. This rate is similar to that reported by Sharma et al. (90%), and somewhat higher than that generally found in the literature (reported to range from 67% to 81%) [31]. Of note, only one out of 35 ND subcentimeter cases was found to be malignant on histopathologic examination. It should be pointed out that the majority of BL cases in our study did not have a surgical follow-up, precluding an evaluation of their ROM. Only 10 out of 144 benign lesions had surgery for the subcentimeter lesions, whilst 182 BL cases from the second series (equal to or larger than 1 cm) had surgery, mostly due to aesthetic/compressive and functional reasons.

Nonetheless, we documented three false negative BL cases in our subcentimeter series. These false negative results might be due to difficulties with aspirating small lesions that resulted in sampling mostly adjacent normal tissue. Nevertheless, the current study has some limitations and biases, including the analysis of a retrospective series, results were obtained from only a single institution, and the performance in cases with suspicious lymph nodes at neck US in 15 cases of our series, leading to the FNAC selection of suspicious subcentimeter nodules. To address these drawbacks, a comparative cohort of nodules greater than 1 cm was included, documenting the presence of one false negative ND case (with FVPTC on follow-up) and three false negative benign nodules histologically classified as one FC, one PTC and one FVPTC.

Some authors and the ATA guidelines attest that FNAC of nodules ≤ 5 mm may yield a high rate of both false positive and ND results [24,25,26,32,45,46]. In our series, we did not find a discrepancy using the cut-off size of ≤5 mm and >5 mm. There were also only 24 (5%) indeterminate proliferations (12 AUS/FLUS and 12 FN/SFN cases) in our subcentimeter series vs. 83 indeterminate cases in the larger nodules. For the subcentimeter series, we identified a 41.6% ROM in the AUS/FLUS category and an even higher 83.3% ROM for FN/SFN. Cavallo et al. found that the ROM for indeterminate cases in their patients was inversely related to nodule size [14]. The vast majority of cases had definitive diagnoses, including 144 (30.3%) benign and 272 (57.2 %) malignant (comprised of 124 SFM and 148 M cases). Hence, these data indicate that there is a higher probability that an US-suspected subcentimeter nodule is malignant. Aydogan et al. similarly demonstrated a 56% malignancy rate in thyroid nodules smaller than 10 mm [29]. We only had one false positive SFM case that was an FA on histopathology, supporting the high dependability of rendering an accurate diagnosis by FNAC in subcentimeter nodules. To support these data, we compared the study dataset with a second series of equal to or larger than 1 cm nodules. The latter series had a prevalence of benign histopathological diagnoses (69.7%) and only 32.3% malignant diagnoses with a low ROM (10.8%) for FN/SFN. For the subcentimeter series, up to 81.5% of our malignant diagnoses were diagnosed as classical PTC, which typically has a favorable prognosis. Nevertheless, we did find that 22 cases were more aggressive PTC variants (TCV, hobnail and solid PTC variants) that were all correctly treated with surgery. On cytology, most of these aggressive variants of PTC were diagnosed as malignant “favoring PTC”. This is in agreement with TBSRTC, which limits diagnosing PTC variants [35]. For NIFTP, none of our FVPTC cases matched the criteria needed to make this diagnosis. The 28 FVPTC cases in this study were histopathologically classified as invasive FVPC because of their infiltrative architectural pattern. For the control cohort of larger nodules, we confirmed the same prevalence of the classical variant of PTC, as well as a few aggressive variants. Furthermore, likely due to their size, in the second series we reported two NIFTP cases, mostly based on the fact that this diagnosis is allowed in nodules larger than 1 cm.

For benign lesions, the comparative cyto-histological correlation between the two cohorts demonstrated a statistically significant correlation, mostly in larger nodules (*p* < 0.0001). The same evaluation for the malignant lesions from the two cohorts was not statistically significant (*p* = 0.51), confirming that the two cohorts did not have significant differences. Furthermore, combining our data with the last edition of the AJCC TNM staging system, we had only 15 cases classified as stage II, confirming the indolent nature of the majority of microcarcinomas. The data are also in line with the general agreement that the majority of subcentimeter lesions are of low risk and amenable to active surveillance rather than warranting immediate surgery.

All of our indeterminate cases (AUS/FLUS and FN/SFN), regardless of size, had both ICC and molecular testing (for *BRAF^V600E^* and *TERT* promoter mutations). For the subcentimeter series, in the AUS/FLUS category, four out of six cases that turned out to be malignant had a concordant positive ICC panel, while the remaining malignant cases showed positivity for only HBME-1. None of the AUS/FLUS cases resulting in a benign histopathological diagnosis had a positive ICC immunopanel, with only two cases out of five demonstrating positivity for only HBME-1. For the subcentimeter FN/SFN cases, a negative ICC immunopanel was found in the two cases with a histopathological diagnosis of FA. All of the FN/SFN cases that had a malignant diagnosis on histopathologic follow-up had at least one positive immunomarker (HBME-1), with 4 out of 10 showing a concordant positive immunopanel. Undoubtedly, immunoreactivity in our series for HBME-1 and galectin-3 supports a diagnosis of malignancy. Only a limited number of mutated cases were identified; 3.1% of cases had *BRAF^V600E^* and none had *TERT* promoter mutations in our series. These results may be attributed to the fact that a large number of subcentimeter carcinomas represent well-differentiated thyroid PTC with an indolent behavior. According to the literature, *TERT* promotor mutations seem to correlate with larger nodules and with more aggressive thyroid carcinomas (including well-differentiated and anaplastic thyroid carcinomas) [47,48,49]. The data from the second series basically overlapped those from the subcentimeter lesions, confirming that regardless of nodular size, the application of an immunopanel may support the morphological diagnosis.

The major limitation of our study is that the evaluation of cases was restricted to nodules that had been submitted for FNAC because of their suspicious US features and/or a previous history of personal/family carcinoma. Nonetheless, as such subcentimeter lesions are more likely to be malignant, the performance of FNAC in these suspicious lesions may contribute to identifying some malignant lesions. According to ATA guidelines, with the contribution of a multi-disciplinary team, the choice for management includes either tailored conservative follow-up or a limited surgical approach.

In summary, combining US features that are useful in characterizing suspected subcentimeter thyroid lesions, FNAC is still recommended to confidently discriminate benign from malignant nodules. Our yield for the subcentimeter group of thyroid nodules determined that FNAC shows a sensitivity of 94.76%, specificity of 99.35%, positive predictive value (PPV) of 99.65%, and negative predictive value (NPV) of 90.65%. The high diagnostic accuracy in FNAC of subcentimeter lesions of the thyroid gland in our series suggested that in presence of suspect US features, the performance of FNAC is advocated even in small nodules. Despite bias due to limited benign subcentimeter nodules, our findings support the implication that thyroid nodules larger than 1 cm are associated with a high incidence of benign lesions on histology. For the minority of cases with an indeterminate diagnosis, ancillary techniques such as ICC proved to be very helpful in refining the cytological diagnosis. Nevertheless, these small nodules should be evaluated by a multidisciplinary team in order to define their best tailored management.

## Figures and Tables

**Table 1:** Summary of clinical/pathologic data.

Number of Cases	<1 cm	≥1 cm
	475 *	606
Gender		
Male	146	239
Female	329	367
Age—mean (range), years	44.38 (16–81)	42.21 (15–79)
Size—mean (range), mm	4.4 (0.3–10)	4 (1–6)
Cytological Diagnostic Categories		
Non-diagnostic	35	29
Benign	144	226
AUS/FLUS	12	54
FN/SFN	12	142
SFM	124	48
M	148	107
Follow-Up		
Surgical follow-up	307	386
Histopathological Diagnoses		
Benign goiter	6	157
Hashimoto thyroiditis	2	5
Follicular adenoma	8	97
PTC and its variants	287	114
MTC	4	6
FC/OFC	0	1–4
NIFTP	0	2
Lymph node metastases *	15	46

AUS/FLUS: atypia of undetermined significance/follicular lesion of undetermined significance; FN/SFN: follicular neoplasm/suspicious for follicular neoplasm; SFM: suspicious for malignancy; M: malignant; FNAC: fine needle aspiration cytology; PTC: papillary thyroid carcinoma; MTC: medullary thyroid carcinoma; FC: follicular carcinoma; OFC: oxyphilic carcinoma; NIFTP: * for the 307 surgical cases.

**Table 2:** US echogeneity stratified according to FNAC diagnostic categories (subcentimeter/equal to or larger than 1 cm nodules).

Bethesda	Hyperechoic (<1 cm/≥1 cm)	Isoechoic (<1 cm/≥1 cm)	Hypoechoic (<1 cm/≥1 cm)	Taller-Than-Wide (<1 cm/≥1 cm)
I (35 cases/29 cases)	5 (14.3%)/1(%)	7 (20%)/8	23 (65.7%)/20	0/0
II (144 cases/226 cases)	5 (3.4%)/0	6 (4.1%)/64	133 (92.3%)/162	0/0
III (12 cases/54 cases)	2 (16.6%)/1	0/8	10 (83.3%)/45	0/0
IV (12 cases/142 cases)	2 (16.6%)/7 (5%)	1 (8.3%)/57	9 (75%)/78	0/0
V (124 cases/48 cases)	3 (2.4%)/1 (%)	9 (7.2%)/1	112 (90.3%)/46	0/0
VI (148 cases/107 cases)	6 (4%)/0	4 (2.7%)/26	138 (93.2%)/79	20 (13.5%)/2

I = non-diagnostic; II = benign; III = AUS/FLUS: atypia of undetermined significance/follicular lesion of undetermined significance; IV = FN/SFN: follicular neoplasm/suspicious for follicular neoplasm; V = SFM: suspicious for malignancy; VI = M: malignant.

**Table 3:** Cytologic/histological correlation for the subcentimeter series of cases.

Diagnosis	Goiter	HT	FA	MTC	PTC	FVPTC	TCV PTC	Warthin PTC	Hobnail PTC	CCV PTC	Solid PTC
ND (2 cases)	1				1						
B (10 cases)	5		2		3						
AUS/FLUS (11 cases)		2	3		2	4					
FN/SFN (12 cases)			2		4	4			2		
SFM (124 cases)			1		105	10	6		2		
M (148 cases)				4	119	10	5	3	2		5

AUS/FLUS: atypia of undetermined significance/follicular lesion of undetermined significance; CCV PTC: columnar cell variant PTC; FA: follicular adenoma; FN/SFN: follicular neoplasm/suspicious for follicular neoplasm; FVPTC: follicular variant of PTC; HT: Hashimoto thyroiditis; M: malignant; MTC: medullary thyroid carcinoma; PTC: papillary thyroid carcinoma; SFM: suspicious for malignancy; TCV PTC: tall cell variant of PTC.

**Table 4:** Cytologic/histological correlation for the centimeter series of cases.

Diagnosis	Goiter	HT	FA	NIFTP	MTC	FC	PTC	FVPTC	TCV PTC	Warthin PTC	Hobnail PTC	CCV PTC	Solid PTC	OFC
ND (4 cases)	3			0				1						
B (182 cases)	145	3	31	0		1	1	1						
AUS/FLUS (37 cases)	4	1	32	0										
FN/SFN (46 cases)	5	1	33	2		3		1						1
SFM (15 cases)			1	0			12	1	1					
M (102 cases)				0	6		89		3	1	2	1		

AUS/FLUS: atypia of undetermined significance/follicular lesion of undetermined significance; CCV PTC: columnar cell variant PTC; FC: follicular carcinoma; FA: follicular adenoma; FN/SFN: follicular neoplasm/suspicious for follicular neoplasm; FVPTC: follicular variant of PTC; HT: Hashimoto thyroiditis; M: malignant; MTC: medullary thyroid carcinoma; OFC: oncocytic carcinoma; PTC: papillary thyroid carcinoma; SFM: suspicious for malignancy; TCV PTC: tall cell variant of PTC.

**Table 5:** Cytologic/histological evaluation of the discrepancies between cytology and histopathology in subcentimeter lesions.

TBRTC Category	Discordant Cases *
Bethesda I (2 cases)	1/2 (50%)
Bethesda II (10 cases)	3/10 (30%)
Bethesda III (11 cases)	6/11 (54.5%)
Bethesda IV (12 cases)	10/12 (83.3%)
Bethesda V (124 cases)	1/124 (0.8%)
Bethesda VI (148 cases)	0/148

I = non-diagnostic; II = benign; III = AUS/FLUS: atypia of undetermined significance/follicular lesion of undetermined significance; IV = FN/SFN: follicular neoplasm/suspicious for follicular neoplasm; V = SFM: suspicious for malignancy; VI = M: malignant. * = 307 cases were analyzed with surgical follow-up, the remaining 168 cases with repeat FNAC.

**Table 6:** Cytologic/histological evaluation of the discrepancies between cytology and histopathology in larger nodules.

TBRTC Category	Discordant Cases *
Bethesda I (4 cases)	1/4 (25%)
Bethesda II (182 cases)	3/182 (1.6%)
Bethesda III (37 cases)	0/37
Bethesda IV (46 cases)	7 */46 (10.8%)
Bethesda V (15 cases)	1/15 (6.6%)
Bethesda VI (102 cases)	0/102

I = non-diagnostic; II = benign; III = AUS/FLUS: atypia of undetermined significance/follicular lesion of undetermined significance; IV = FN/SFN: follicular neoplasm/suspicious for follicular neoplasm; V = SFM: suspicious for malignancy; VI = M: malignant; * = 2 NIFTP were included as malignant diagnoses.

**Table 7:** ICC evaluation performed on 107 cases out of 475.

Bethesda Category	H+/G+	Discordant	H−/G−	Calcitonin
I (35 cases)	0	0	0	0
II (144 cases)	0	0	39 (27%)	0
III (12 cases)	4 (33.3%)	5 (41.6%)	3 (25%)	0
IV (12 cases)	4 (33.3%)	6 (50%)	2 (16.6%)	0
V (124 cases)	42 (33.8%)	3 (2.4%)	0	0
VI (148 cases)	10 (6.7%)	0	0	4 (2.7%)

I = non-diagnostic; II = benign; III = AUS/FLUS: atypia of undetermined significance/follicular lesion of undetermined significance; IV = FN/SFN: follicular neoplasm/suspicious for follicular neoplasm; V = SFM: suspicious for malignancy; VI = M: malignant; H = HBME-1; G = galectin-3.

**Table 8:** ICC evaluation performed on 122 control cases out of 386.

Bethesda Category	H+/G+	Discordant	H−/G−	Calcitonin
I (4 cases)	0	0	0	0
II (182 cases)	0	0	5 (2.7%)	0
III (37 cases)	3 (8.1%)	8 (21.6%)	26 (70.2%)	0
IV (46 cases)	5 (10.8%)	16 (34.7%)	25 (54.3%)	0
V (15 cases)	12 (80%)	2 (13.3%)	1 (6.6%)	0
VI (102 cases)	15 (14.7%)	0	0	4 (3.9%)

I = non-diagnostic; II = benign; III = AUS/FLUS: atypia of undetermined significance/follicular lesion of undetermined significance; IV = FN/SFN: follicular neoplasm/suspicious for follicular neoplasm; V = SFM: suspicious for malignancy; VI = M: malignant; H = HBME-1; G = galectin-3.

**Table 9:** Molecular testing for *BRAF^V600E^* and *TERT* promoter mutations in the subcentimeter series.

Bethesda Category	*BRAF*+	*BRAF*−	TERT Mutated
I (35 cases)	0	0	0
II (144 cases)	0	0	0
III (12 cases)	0	12 (100%)	0
IV (12 cases)	1 (8.3%)	11 (91.6%)	0
V (124 cases)	9 (7.2%)	10 (8%)	0
VI (148 cases)	5 (3.3%)	10 (6.7%)	0

I = non-diagnostic; II = benign; III = AUS/FLUS: atypia of undetermined significance/follicular lesion of undetermined significance; IV = FN/SFN: follicular neoplasm/suspicious for follicular neoplasm; V = SFM: suspicious for malignancy; VI = M: malignant.

**Table 10:** Molecular testing for *BRAF^V600E^* and *TERT* promoter mutations in larger nodules.

Bethesda Category	*BRAF*+	*BRAF*−	TERT Mutated
I (4 cases)	0	0	0
II (182 cases)	0	0	0
III (37 cases)	0	37 (100%)	0
IV (46 cases)	1 (2.1%)	13 (28.2%)	0
V (15 cases)	6 (40%)	9 (60%)	0
VI (102 cases)	2 (1.9%)	28 (27.4%)	0

I = non-diagnostic; II = benign; III = AUS/FLUS: atypia of undetermined significance/follicular lesion of undetermined significance; IV = FN/SFN: follicular neoplasm/suspicious for follicular neoplasm; V = SFM: suspicious for malignancy; VI = M: malignant.

## Data Availability

The preliminary data of this paper have been presented as poster presentation at the USCAP meeting in 2019.
